# Greenhouse and field evaluation of transgenic poplars with modified gibberellin metabolism and signaling genes

**DOI:** 10.1186/1753-6561-5-S7-O22

**Published:** 2011-09-13

**Authors:** Venkatesh Viswanath, Cathleen Ma, Elizabeth Etherington, Palitha Dharmawardhana, David W Pearce, Stewart B Rood, Victor B Busov, Steven H Strauss

**Affiliations:** 1 Department of Forest Ecosystems and Society, Oregon State University, 321 Richardson Hall, Corvallis, OR 97331, USA; 2 Department of Botany and Plant Pathology, Oregon State University, 2082 Cordley Hall, Corvallis, OR 97331, USA; 3 Department of Biological Sciences, University of Lethbridge, 4401 University Dr. W, Lethbridge, AB T1K 3M4, Canada; 4 Department of Biological Sciences, University of Lethbridge, 4401 University Dr. W, Lethbridge, AB T1K 3M4, Canada; 5 School of Forest Resources and Environmental Science, Forestry and Wood Products Building, Michigan Technological University, 1400 Townsend Dr. Houghton, MI 49931, USA

## Summary

Although there have been many observations of transgenes leading to improved plant growth in greenhouse experiments, there is a paucity of data that have been validated by multiple greenhouse experiments or field studies. Genes that encode regulators of gibberellin metabolic pathways were among the first reported to improve biomass growth in trees, and thus were the logical focus of this study. Poplars were transformed with eight different transgenic constructs designed to modify gibberellin biosynthesis and/or signaling. Dramatic improvements in growth rate were observed for several constructs under greenhouse conditions. However, these were often not seen in repeated greenhouse studies, and were statistically unconfirmed for most constructs in the field. Our results underline the importance of multiple greenhouse and field trials during studies with transgenes that modify hormonal homeostasis.

## Genes studied

The genes under investigation in this study were *GA20-oxidase*, *PHOR1-1*, and *SPINDLY*. GA20-oxidase catalyzes the conversion of C_20_-gibberellins to C_19_-gibberellins through a series of sequential oxidation steps using 2-oxoglutarate as a co- substrate [1]. This enzyme was first isolated from pumpkin cotyledons [2], and is involved in the penultimate step in the production of bioactive gibberellins. The *PHOR1-1* gene is a positive regulator that was first isolated from leaves of potato plants subjected to a short day photoperiod (SD) [3]. Altered feedback regulation was observed in transgenic plants produced by antisense inhibition of this gene, indicating the role of the PHOR1-1 protein as an intermediate in the gibberellin signaling pathway. *SPINDLY* (*SPY*) is a gene that codes for an O-linked N-acetylglucosamine transferase [4]. In *Arabidopsis*, it has been shown to inhibit GA signaling, and thus is considered a negative regulator of GA signaling [5].

## Methods

Combinations of the three different genes and seven different promoters were used (Table 1).

**Table 1:** Summary of constructs used in the transformation experiments

Promoter	Gene	Terminator	Number of events studied (GH/Field)	Total number of trees (GH/Field)
PtGA20ox7	*PtGA20ox7*	PtGA20ox7	38/35	345/278
PtPHOR1	*PtPHOR1*	PtPHOR1	26/3	327/24
RGL1-1	*PtGA20ox2-2*	NOS	34/17	444/83
GA2ox1	*PtGA20ox2-2*	NOS	30/15	425/75
Β-expansin	*PtGA20ox2-2*	NOS	24/0	120/0
PtCESA1	*PtGA20ox2-2*	NOS	16/0	80/0
35S	*AtSPY*	OCS	33/12	163/48
35S	*HvSPY*	OCS	33/12	162/48

In addition to constitutive promoters, we used native promoters of poplar genes which were identified by screening whole-genome microarray expression data. Plants transformed with all eight constructs were grown in the greenhouse, while those transformed with six of these constructs were also grown in the field. For four constructs, the greenhouse trials were repeated at least once. In all, 2,066 trees were studied in the greenhouse (286 independent transformations) and 556 trees (94 independent transformations) were studied in the field. Transgenic and non-transgenic controls were included in both the greenhouse and field studies. Height and diameter measurements were recorded in both the greenhouse and the field, and shoot and root biomass were measured on the greenhouse-grown plants. Levels of bioactive gibberellins were also measured on a subset of plants transformed with the two cisgenes (a gene having driven by its own promoter and ending with its own terminator), as well as in plants that had the RGL and the GA2-oxidase promoters driving the *PtGA20-oxidase* gene.

## Results and discussion

**Figure 1:**
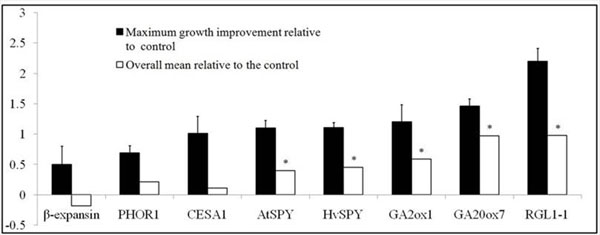
Results from greenhouse trials of transgenic plants. The constructs are denoted by the promoter used, except in the case of *SPY* genes. Dark bars show the maximum fractional rate improvement (best two transgenic events from best greenhouse experiment) compared to controls. Open bars show the mean fractional growth rate improvement for all transgenic events in that greenhose experiment. Brackets denote one standard error. A “*” above an open bar indicates statistically significant difference between the transgenic and control groups.

When considering the best growth rate improvements for constructs that had repeated greenhouse trials, the maximum growth rate improvement seen (the mean of the best two events relative to the control) varied from 50 to 220% i.e., a fractional growth rate improvement ranging from 0.5 to 2.2 (Figure 1).

However, results were not consistent between the different greenhouse trials. The best events showed more than 100% in maximum growth rate improvement (fractional growth rate improvement > 1) in six different greenhouse experiments (Fig. 1); however, these events did not have a similar extent of improvement when the experiments were repeated. Five out of eight constructs produced a statistically significant improvement in growth rate (Figure 1).

Only one out of the six constructs tested in the field produced a statistically significant improvement in volume index, and this was for the RGL promoter driving the *PtGA20-oxidase* gene. The GA20-oxidase and the PHOR1 cisgenic plants, when grown in the greenhouse, had substantial increases (73-133%) in the levels of bioactive gibberellins in developing shoots compared to control plants, however, there was no significant correlation between gibberellin levels and volume index of each event. The levels of the gibberellin biosynthetic precursors were also measured and found to be greater than the bioactive gibberellin level, suggesting feedback inhibition—a possible mechanism for the lack of association with growth. Our results suggest that physiology-modifying transgenes strongly interact with growth environments. This implies that, as in conventional plant breeding, greenhouse results are of limited value and field trials are essential for evaluation of varieties with improved productivity.
